# Utilization of Low-Grade Limestone and Solid Waste for the Preparation of High-Belite Portland Cement

**DOI:** 10.3390/ma18112641

**Published:** 2025-06-04

**Authors:** Jiapeng Duan, Yu Zhang, Suwei Xia, Zian Geng, Wenbo Xin

**Affiliations:** State Key Laboratory of Materials-Oriented Chemical Engineering, College of Materials Science and Engineering, Nanjing Tech University, Nanjing 211816, China

**Keywords:** low-grade limestone, high belite, Portland cement, lower CO_2_ emission

## Abstract

In this study, high-belite Portland cement clinker was successfully prepared by using low-grade limestone and solid-waste calcium carbide slag and steel slag, achieving resource utilization while reducing CO_2_ emissions caused by raw materials decomposition in the cement industry. Using X-ray diffraction, microscopic images, thermogravimetric analysis, and differential scanning calorimetry, the physicochemical reaction process, phase composition, and microscopy of clinker were studied. The results indicated that the high-belite Portland cement clinker can be successfully produced at 1340 °C for 1 h with a belite content of 58.6% and an alite content of 24.2% when the composition of raw material was suitable. Meanwhile, the content of high-reactive-phase α-C_2_S can reach 1.4%. Via microscopic viewing, C_2_S and C_3_S were interphase distributed and well developed. In this study, the CO_2_ emission of the prepared high-belite Portland cement clinker was 54.67% lower than that of ordinary Portland cement clinker. All the above results confirm that high-belite Portland cement clinker can be produced using low-grade limestone and solid wastes, which can significantly reduce CO_2_ emission during Portland clinker production and promote an innovative approach to the cement industry.

## 1. Introduction

Based on global cement production in 2023, China remains the world’s largest cement producer, accounting for about 52% of the global total. According to the national statistics, the total cement production in China reached 2.02 billion tons in 2023 [1], ranking the first in the world for almost 40 years. Normally, there are 0.6 tons of CO_2_ emission for each ton of Portland cement clinker production, resulting a total of 1.21 billion tons of CO_2_ emission in the cement industry in China in 2023. In 2023, China’s total carbon emission amounted to 12.6 billion tons [2], of which cement clinker production contributed 9.6% to the overall emission. The low-carbon development of the cement industry is crucial for China to achieve carbon neutrality. Reducing CO_2_ emission during the calcination process of Portland cement is considered one of the best pathways to decrease overall CO_2_ emission in cement production. High-belite cement (HBC) is a type of Portland cement characterized by belite (C_2_S) as its primary mineral phase, with the C_2_S content exceeding 40%. As belite Portland cement, it is a Portland clinker mainly composed of belite mineral phases. In cement clinker, compared with alite, belite has a lower early hydration activity and mainly contributes to the later-phase strength, while alite mainly determines the early strength of cement. In the cement industry, HBC typically contains 40–50% C_2_S and 25–30% alite (C_3_S) [3].

The substitution of HBC for ordinary Portland cement (PC) can significantly lower the quality requirements for raw materials, enabling the use of low-grade limestone (LGL) in place of high-grade limestone. Additionally, CO_2_ emission is significantly reduced. In the aspects of phase mineralization of Portland cement clinker, the formation enthalpy of C_3_S is 1810 kJ/kg, while the enthalpy of C_2_S is 1350 kJ/kg [4]. Moreover, the calcination temperature for HBC is lower than that of PC [5]. Consequently, this reduced calcination temperature leads to decreased heat consumption during clinker production, thereby mitigating the environmental impact associated with cement manufacture. Moreover, HBC exhibits low heat of hydration, superior high-temperature stability, exceptional resistance to chemical attack, and a gradual early strength development, followed by a significant long-term strength gain.

HBC can utilize low-grade raw materials or solid waste as substitutes for high-grade limestone owing to its lower requirements for raw materials quality. This provides a new solution for the utilization of LGL and solid wastes. Jihane Moudar et al. [6] successfully prepared belite using waste glass and shells instead of traditional raw materials. Their study demonstrated that the 90-day hydration strength of cement mortar reached 30.05 MPa after blending the synthesized C_2_S with shellac and gypsum. M.K. Enríquez et al. [7] further investigated the use of paper sludge, cement kiln dust, and rice husk ash as complete substitutes for natural raw materials in belite cement clinker production. Their research revealed that HBC clinker could be manufactured using 20–25% paper sludge, 60–69% cement kiln dust, and 9–11% rice husk ash. These studies collectively demonstrate the feasibility and environmental benefits of incorporating low-quality raw materials and waste streams into HBC production, aligning with global sustainability goals while maintaining or enhancing cement properties. In addition, the water–cement ratio of cement paste has a significant impact on rheological and time-varying properties, which, in turn, has a huge influence on its mechanical properties [8,9,10,11,12].

Calcium carbide slag, a by-product generated from the hydrolysis of calcium carbide to produce acetylene gas, poses significant environmental hazard when accumulated in large quantities. It has been extensively utilized in various sectors, including building materials, chemical engineering, and pollution abatement [13]. The primary constituent of calcium carbide slag is Ca(OH)_2_, which can be thermally decomposed into CaO at elevated temperatures and subsequently utilized as a raw material in cement production. Relative to limestone, calcium carbide slag exhibits lower decomposition heat and does not release CO_2_ during the process, thereby offering significant advantages in terms of energy conservation and emission reduction [14]. Moreover, 1 ton of calcium carbide slag can substitute for 1.28 tons of limestone, thereby reducing CO_2_ emission by 0.56 tons. This approach effectively achieves the objective of “treating waste with waste” [15]. Yifan Gao et al. [16] prepared belite cement using solid waste materials, including calcium carbide slag, silica fume, and laterite. The Ca^2+^ ions in the calcium carbide slag can be substituted by other ions present in these waste materials. The resulting belite cement exhibits superior long-term strength compared to ordinary Portland cement. Low-grade limestone, a by-product of limestone mining operations, has gained increasing attention for resource utilization as high-grade limestone resources gradually deplete. The resource utilization of LGL has become one of the important directions in the development of the cement industry. Tamma VR et al. [17] prepared limestone-based cement by partially substituting LGL for cement clinker. Research findings indicate that when the substitution ratio is 15%, the performance of the resulting limestone-based cement is equivalent to that of 43-grade ordinary Portland cement. Liu Zhenhe et al. [18] successfully developed cement clinker utilizing low-grade limestone, achieving a 28-day compressive strength exceeding 55 MPa.

**Figure 1:**
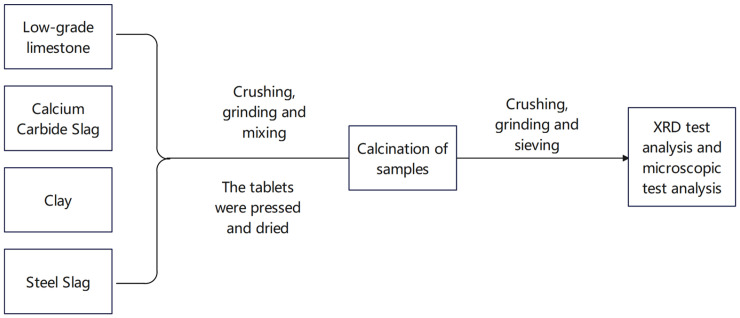
Technical roadmap.

The aforementioned research demonstrates that belite cement clinker can be successfully synthesized from low-grade raw materials and solid waste. In this study, high-belite Portland cement clinker was produced using calcium carbide slag, steel slag, LGL, and clay. The reactivity of low-grade limestone was enhanced via mechanical activation. This approach not only facilitates the resource utilization of LGL and solid wastes but also significantly reduces CO_2_ emission during the cement production. Furthermore, high-belite Portland cement exhibits superior chemical properties and a broader range of applications compared to conventional Portland cement. The technical roadmap of this article is shown in Figure 1 as follows:

## 2. Experimental Section

### 2.1. Raw Materials

**Figure 2:**
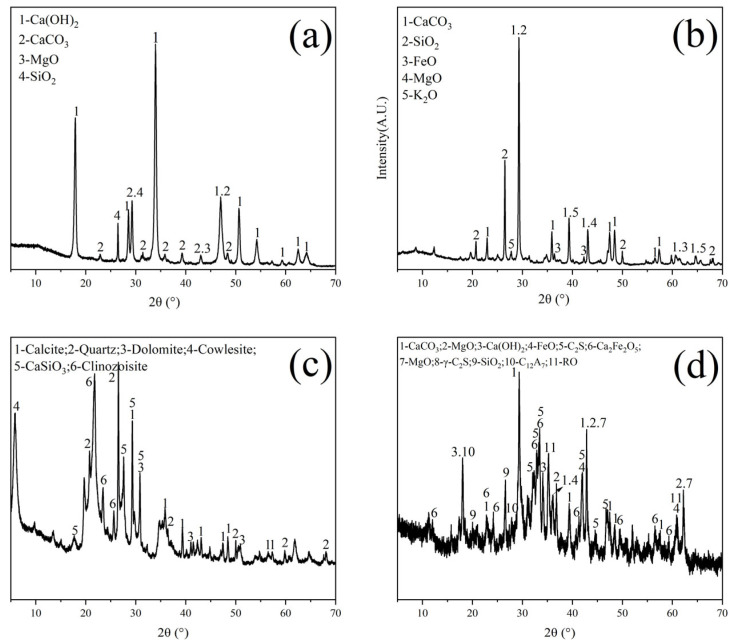
XRD diagram of raw materials. (**a**) Calcium carbide slag, (**b**) low-grade limestone, (**c**) clay, and (**d**) steel slag.

**Figure 3:**
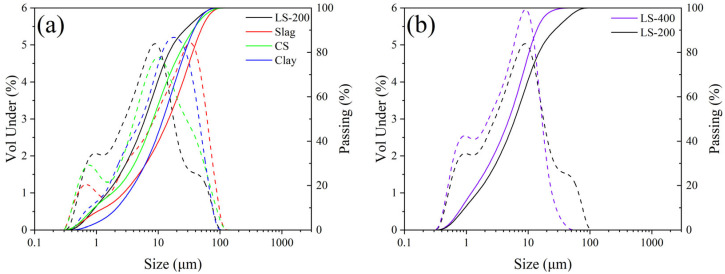
Particle size distribution of raw materials. Solid line: passage rate; dashed line: volume ratio. (**a**) Particle size distribution of raw materials, (**b**) Particle size distribution of different limestone.

The raw materials utilized in this study include LGL (Conch Cement, Huainan, China), calcium carbide slag (Conch Cement, Huainan, China), clay (Shijiazhuang, China), and steel slag (Maanshan, China). Table 1 provides the chemical compositions of the raw materials. Figure 2 illustrates the X-ray diffraction (XRD) patterns of the raw materials, where the main substances in calcium carbide slag are Ca(OH)_2_ and CaCO_3_. For low-grade limestone, the main substances are CaCO_3_ and SiO_2_. In clay, the main components are SiO_2_ and silicate minerals. The composition of steel slag is relatively complex. Besides CaCO_3_ and Fe_2_O_3_, there are also mineral phases from clinker, such as C_2_S, etc. The particle size distributions of the raw materials are shown in Figure 3a, and the median particle sizes of the LGL, calcium carbide slag, clay, and steel slag are 6.113 μm, 8.278 μm, 11.986 μm, and 14.785 μm, respectively. Additionally, the LGL underwent graded grinding to pass through 200-mesh (74 μm) and 400-mesh (34 μm) square hole sieves, designated as LS-200 and LS-400, and their particle size distributions are shown in Figure 3b. The median particle sizes of LS-200 and LS-400 are 6.133 μm and 4.907 μm, respectively.

**Figure 4:**
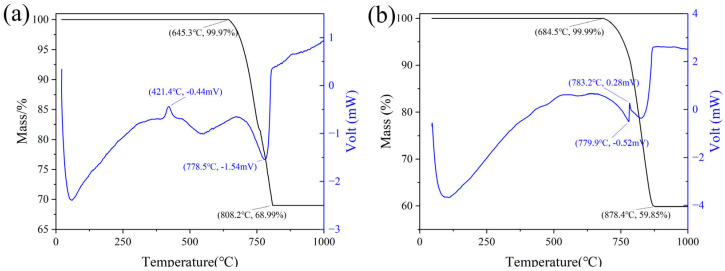
Comparison of TG−DSC between low−grade limestone and common limestone. (**a**) low−grade limestone, (**b**) common limestone.

LGL contains a higher proportion of SiO_2_ than common limestone and exhibits differences in reactivity during calcination with CaCO_3_. Thermogravimetric (TG) and differential scanning calorimetry (DSC) analyses of low-grade limestone and common limestone are shown in Figure 4, where it was observed that low-grade limestone releases less CO_2_ during thermal decomposition. The DSC curve of common limestone shows more pronounced peaks and valleys, indicating a higher energy barrier for calcite decomposition [19]. The impurities present in LGL are beneficial to reduce the decomposition temperature, which starts at 645.3 °C, while the decomposition temperature of common limestone is 684.5 °C.

The mineral-phase composition in cement clinker can be controlled by adjusting three critical parameters: the limestone saturation factor (KH), the silica modulus (SM), and the alumina modulus (IM). By optimizing the values of these parameters, the initial mineral composition of the cement clinker can be initially set. The calculation formulas are as follows [20]:

(1) $$KH=\frac{\mathrm{CaO}-1{.65\mathrm{Al}}_{2}O_{3}-0.35{\mathrm{Fe}}_{2}O_{3}}{2.8{\mathrm{SiO}}_{2}}$$

(2) $$SM=\frac{{\mathrm{SiO}}_{2}}{{\mathrm{Al}}_{2}O_{3}{+\mathrm{Fe}}_{2}O_{3}}$$

(3) $$IM=\frac{{\mathrm{Al}}_{2}O_{3}}{{\mathrm{Fe}}_{2}O_{3}}$$

The chemical composition of the raw materials and the three parameters are illustrated in Table 2. This study aims to incorporate LGL and calcium carbide slag, among other solid wastes, into cement clinker production. By precisely adjusting the proportions of each raw material, a set of parameters are prepared (KH = 0.70–0.90, SM = 2.5 ± 0.1, and IM = 1.2 ± 0.1). Specifically, high-belite Portland cement clinker is produced by systematically varying the KH value, while the values of SM and IM are constant.

**Figure 5:**
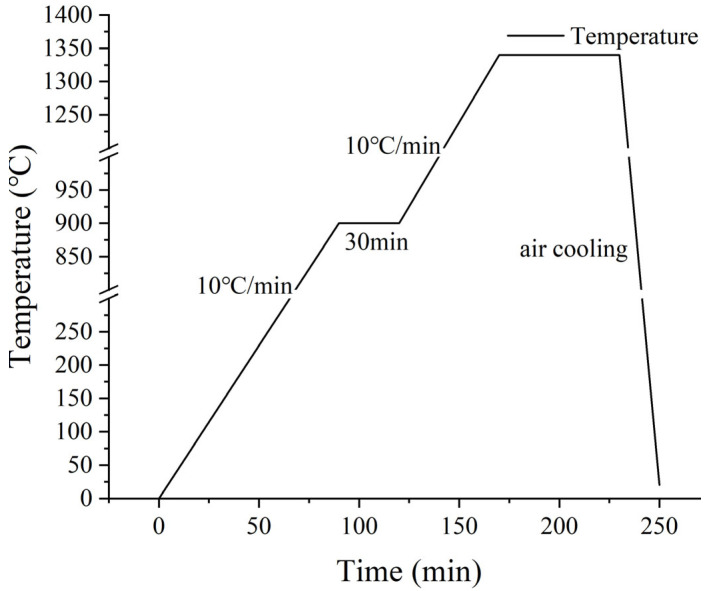
Calcination process.

Samples are prepared with the specified ratio according to Table 2. For each specimen, 50 g of the mixture was combined with 8% deionized water and thoroughly blended for 12 h. Subsequently, a tablet press was utilized to form a square piece with a thickness of about 4 cm. The prepared sample was then placed in an oven at 105 °C for 24 h to dry. The dried square pieces were then transferred to a box-type resistance furnace for sintering. The temperature was increased at a rate of 10 °C per minute, held at 900 °C for 30 min, and further heated to the target temperature with subsequent insulation. The detailed sintering procedure is illustrated in Figure 5.

### 2.2. Measurement Methods

The XRD measurements were conducted using a SmartLabTM diffractometer from Rigaku, Tokyo, Japan. The instrument was equipped with a copper target (CuKα, λ = 0.154 nm), and the data were collected from 5° to 70° with a step size of 0.01° at 5°/min. The X-ray tube operated at 40 kV and 40 mA. Prior to the XRD tests, the samples were milled to a particle size of ≤45 μm to minimize preferred orientations.

Mineral-phase identification was performed using Search Match software (Version 4.0), which allows for rapid and accurate determination of the corresponding mineral PDF cards. Table 3 presents the CIF files corresponding to the clinker mineral phases used in this experiment. Quantitative analysis of the cement clinker was performed using the Rietveld method with High Score Plus. During the calculation process, the weighted profile residual (Rwp) was minimized to ≤10.0%, and the Goodness of Fit (G) was maintained at ≤2.0 [21].

**Table 3:** ICSD numbers of mineral phase.

Phase	ICSD Number
M1-C_3_S	81100 [22]
M3-C_3_S	94742 [23]
α-C_2_S	81099 [24]
β-C_2_S	81096 [25]
γ-C_2_S	81095 [26]
C_3_A	1841 [27]
C_4_AF	9197 [28]
CaO	75785 [29]

TG-DSC curves were conducted using a STA 449F5 instrument (NETZSCH, Selb, Germany). The heating rate was 10 °C/min, with a temperature range of 20–1500 °C under an air atmosphere for the initial tests. For hydrated samples, the same heating rate was applied within a temperature range of 20–1000 °C under a nitrogen (N_2_) atmosphere.

Microscopic images were captured on internal cross-sections of the samples with a Gemini 300 scanning electron microscope (ZEISS, Oberkochen, Germany) at an accelerating voltage of 3.0 kV. And the samples were sprayed with gold before the experiment

## 3. Results and Discussion

**Figure 6:**
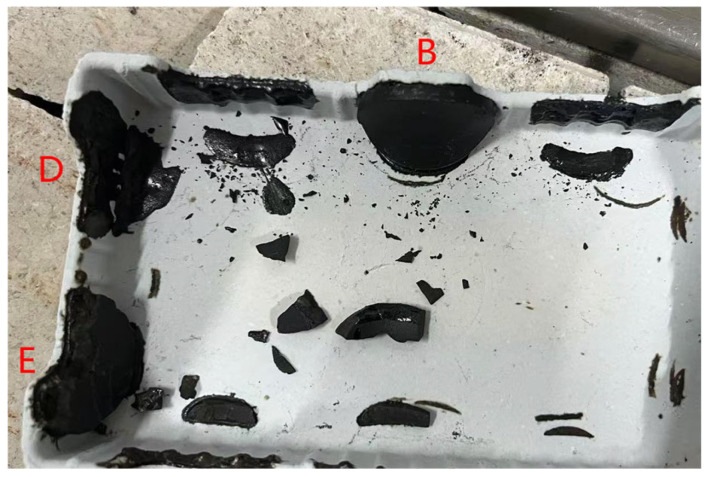
Low-KH clinker sample photo (B, D, E represent clinker with a low KH ratio).

**Figure 7:**
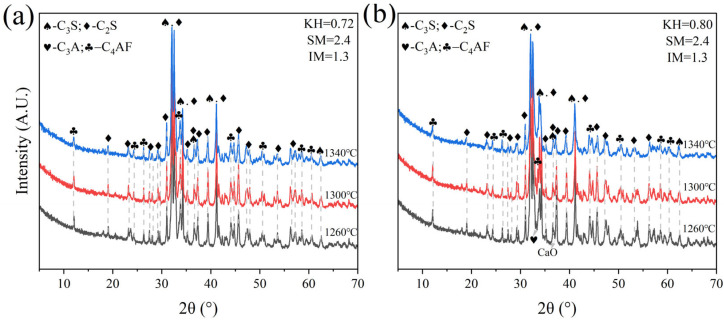
XRD analysis patterns of samples A and E. (**a**) sample A, (**b**) sample E.

**Figure 8:**
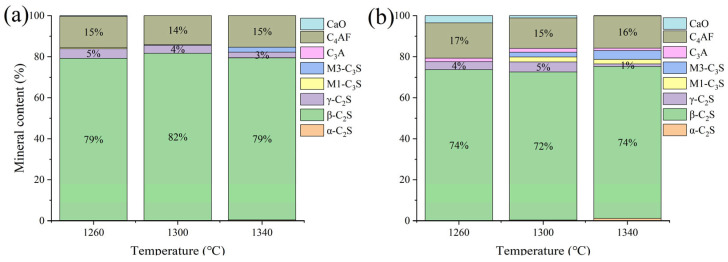
The proportion of major mineral components in samples A and E. (**a**) sample A, (**b**) sample E.

Low-KH (0.72–0.80) clinker was calcined at temperatures of 1260 °C, 1300 °C, 1340 °C, and 1380 °C, each with a sintering time for 1 h. It was observed that a melting phenomenon occurred during calcination at 1380 °C (Figure 6). Quantitative XRD analysis (Rwp ≤ 10%, G ≤ 2.0) of samples calcined below 1340 °C revealed that the primary mineral phase was C_2_S, comprising 70–80%, with C_3_S present in trace amounts (0–10%), as shown in Figure 7 and Figure 8. Given the predominance of C_2_S and the relatively low content of C_3_S, the resulting clinker exhibits insufficient strength for practical applications and is, therefore, unsuitable for manufacturing purposes.

**Figure 9:**
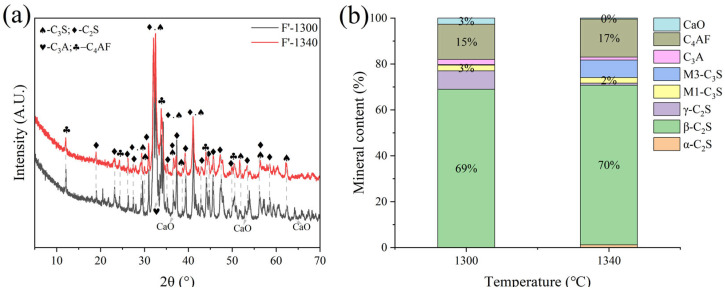
XRD analysis and mineral composition ratios of sample F. (**a**) XRD of sample F, (**b**) mineral content of sample F.

Sample F was subjected to calcination at temperatures of 1300 °C, 1340 °C, and 1380 °C with a sintering time of 1 h. It was observed that calcination at 1380 °C resulted in significant melting and obvious adherence to the crucible. Calcination at 1300 °C and 1340 °C proceeded normally. XRD analysis (Figure 9) revealed that at 1300 °C, a substantial amount of f-CaO remained unreacted, with C_2_S comprising 77% of the main mineral phase and C_3_S only 2.7%. At 1340 °C, the main mineral phase was also predominantly C_2_S (77%), with a C_3_S content of 2.7%.

**Figure 10:**
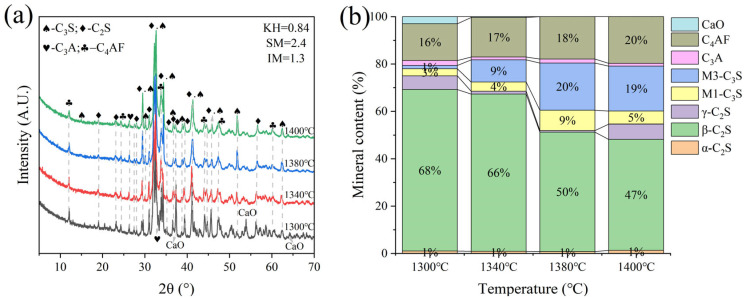
XRD analysis and mineral composition ratios of sample G. (**a**) XRD of sample G, (**b**) mineral content of sample G.

**Figure 11:**
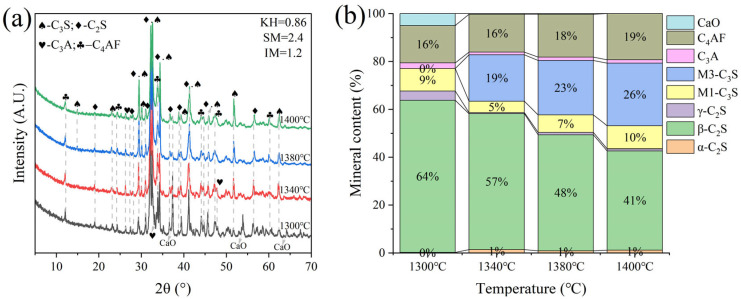
XRD analysis and mineral composition ratios of sample H. (**a**) XRD of sample H, (**b**) mineral content of sample H.

**Figure 12:**
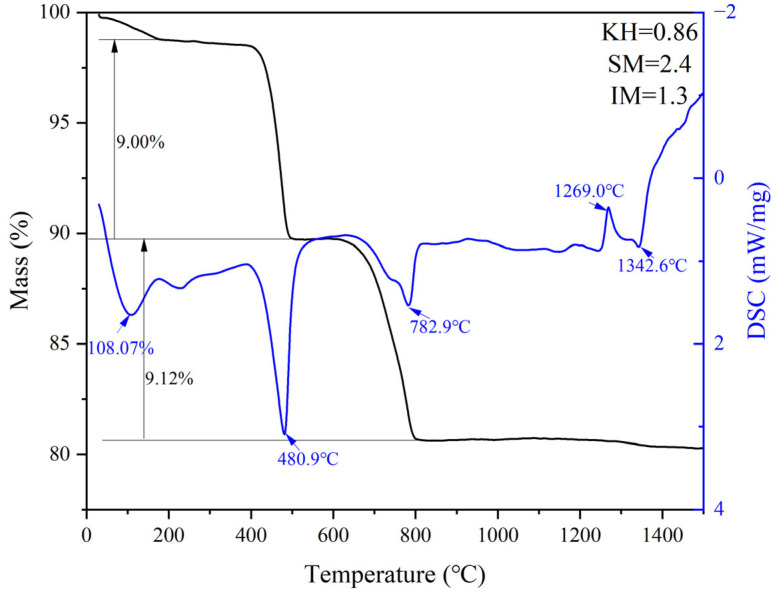
TG-DSC curves of sample H.

G and H were calcined at temperatures of 1300 °C, 1340 °C, 1380 °C, and 1400 °C for 1 h. The XRD and mineral composition of samples H and G are shown in Figure 10 and Figure 11. Calcination at 1300 °C resulted in a significant amount of f-CaO and pulverization, while the diffraction peaks corresponding to f-CaO disappeared at 1340 °C. The quantitative analysis shows that the C_2_S in the clinker was 68.5%, and the C_3_S was only 13.3% when the sample G was calcined at 1340 °C. C_2_S and C_3_S were 51.9% and 28.5%, respectively at 1380 °C, but the C_3_A content was slightly higher at 1.7%. The clinker at 1400 °C was 54.7% C_2_S and 24.5% C_3_S, but the content of γ-C_2_S was too high. The H sample exhibited 58.6% C_2_S and 24.2 % C_3_S at 1340 °C, 50.4% C_2_S and 30.1% C_3_S at 1380 °C, and 43.4% C_2_S and 35.8% C_3_S at 1400 °C. TG-DSC analysis of the H sample (Figure 12) showed two significant mass losses of 9.00% and 9.12% at temperatures of 368–513 °C and 606–833 °C, respectively, corresponding to the decomposition of Ca(OH)_2_ and CaCO_3_. The DSC curves revealed an endothermic peak at 108.07 °C due to the evaporation of free water in the raw material, as well as peaks at 480.9 °C and 782.9 °C, which corresponded to the decomposition of Ca(OH)_2_ and CaCO_3_, consistent with the TG data. Additionally, an exothermic peak at 1269.0 °C was observed, likely resulting from the thermal effect of C_2_S formation during solid-phase precipitation, while a peak at 1342.6 °C was attributed to the sintering of the molten phase, representing the thermal effect of C_3_S formation [30]. XRD and mineral-phase composition analysis concluded that more C_3_S would be produced in sample H at the same calcination temperature, which is favorable for clinker strength. In addition, more calcium carbide slag could be used in sample H mixing to reduce CO_2_ emission more effectively.

**Figure 13:**
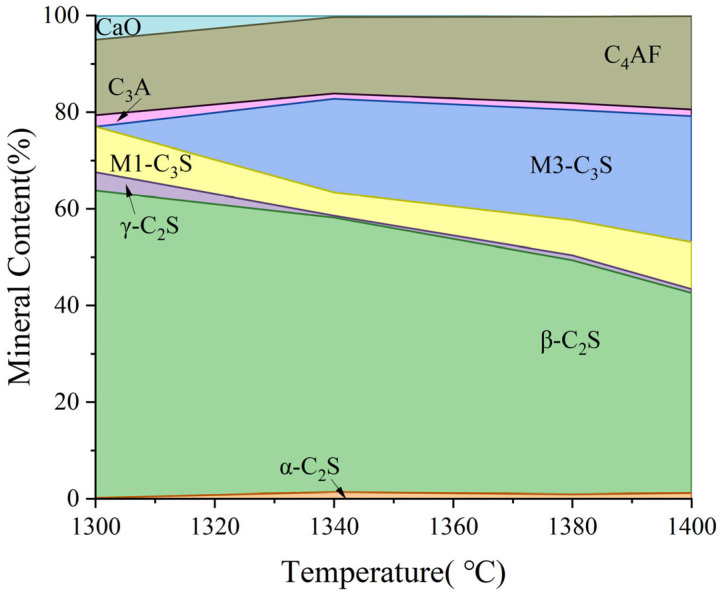
Effect of calcination temperature on mineral-phase formation.

As shown in Figure 13, there is unreacted CaO in the clinker at a low calcining temperature. At this time, the content of β-C_2_S in the clinker accounts for more than 60%, and a large amount of β-C_2_S makes it easy to produce more γ-C_2_S during cooling. Meanwhile, the C3S in the clinker calcined at 1300 °C is of the M1 type, which is related to the presence of trace mineralization ions in the raw material, which can promote the transformation of the M3 type to the M1 type. With the increase in calcination temperature, CaO in clinker reacts with C_2_S to form C_3_S, M3-C_3_S begins to appear, and the content gradually increases, but the change in M1-C_3_S content floats less, which is limited by the content of mineralization ions. When the calcination temperature was increased to 1380 °C, the content of C_3_S in clinker was 30%. Trace mineralization ions in the raw material have a positive effect on the calcination of clinker, which can promote the generation of highly active minerals, such as M1-C_3_S and α-C_2_S. In addition, the increase in calcination temperature promotes the conversion of C_2_S to C_3_S, and the CaO content in clinker is also reduced. However, the clinker intermediate phases, constituting C_4_AF and C_3_A generation, were basically unchanged.

**Figure 14:**
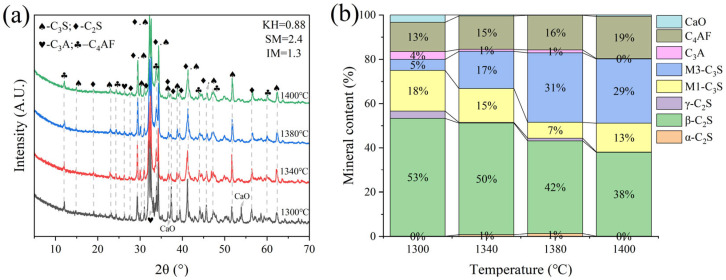
XRD analysis and mineral composition ratios of sample I. (**a**) XRD of sample I, (**b**) mineral content of sample I.

**Figure 15:**
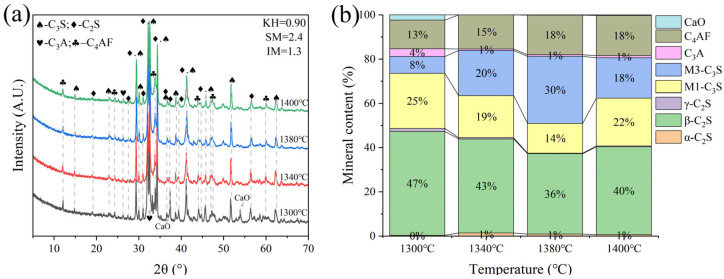
XRD analysis and mineral composition ratios of sample J. (**a**) XRD of sample J, (**b**) mineral content of sample J.

For samples I and J, calcination experiments were performed at temperatures of 1340 °C, 1380 °C, and 1400 °C, each with a one-hour sintering period. XRD qualitative and quantitative analyses indicated that the C_3_S content in cement clinker calcined at 1340 °C exceeded 30% (Figure 14 and Figure 15). However, due to an imbalanced mineral-phase composition, characterized by a lower KH value and an excessively high IM value, the formation of C_3_S from C_2_S and CaO was hindered. The high IM values resulted in increased liquid-phase viscosity, complicating mass diffusion and thereby reducing the contribution of C_3_S to the clinker’s mineral-phase composition [31]. It was determined that the clinker prepared with the KH = 0.86 ratio is more suitable for use in production. Additionally, insulation time experiments were conducted on sample H to determine the optimal sintering duration.

**Figure 16:**
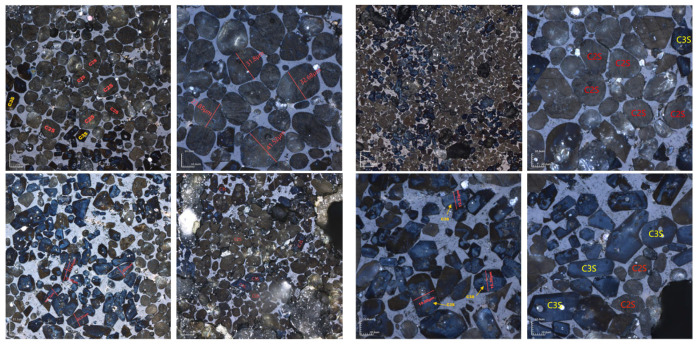
Microscopic images of sample H sintered at 1340 °C and 1380 °C for 1 h. (**Left**): 1340 °C, (**Right**): 1380 °C.

Due to the minimal difference in mineral-phase composition between calcination temperatures of 1340 °C and 1380 °C, microscopic image analysis was conducted at both temperatures to further determine the optimal calcination temperature. The figure below shows the microscopic image analysis of cement clinker prepared at 1340 °C and 1380 °C with KH = 0.86 (Figure 16). For the sample calcined at 1340 °C with a one-hour sintering period, the main mineral phases were C_2_S and C_3_S, with C_2_S particles surrounded by the liquid phase [32]. The C_2_S mineral phase had an average particle size of approximately 30 μm, while the C_3_S mineral phase had an average particle size of about 20 μm and a length-to-width ratio of 2.5. In contrast, the C_3_S mineral phase significantly increased in samples calcined at 1380 °C. Quantitative analysis indicated that calcination at 1340 °C was more appropriate.

**Figure 17:**
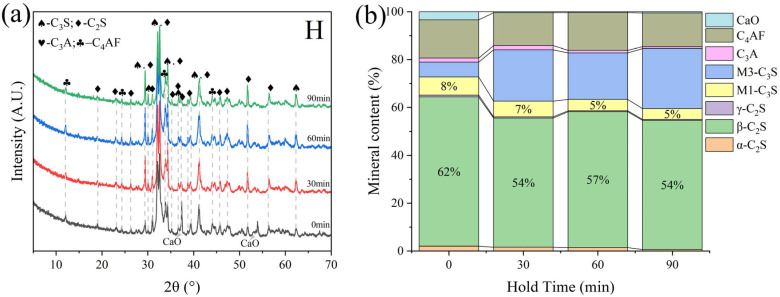
XRD and mineral content of sample H under varying sintering time conditions. (**a**) XRD of sample H, (**b**) mineral content of sample H.

**Figure 18:**
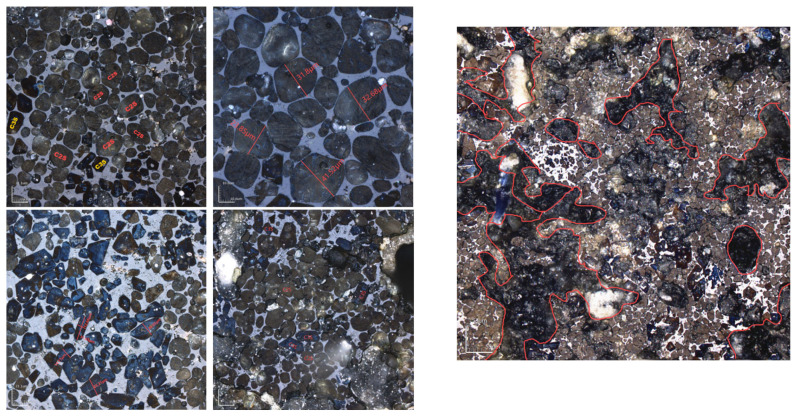
Microscopic images of sample H sintered at 1340 °C for 30 min and 1 h. (**Left**): 1 h; (**Right**): 30 min.

Different sintering time experiments were conducted for sample H under the desired ratio, with sintering times of 0 min, 30 min, 60 min, and 90 min. XRD and microscopic analyses revealed distinct f-CaO diffraction peaks, with quantitative analysis indicating a higher f-CaO content at a calcination temperature of 1340 °C when the sintering time was reduced to 0 min in Figure 17. Additionally, the C_3_S content significantly increased at a sintering time of 90 min. The mineral-phase composition at 30 min closely matched that observed at 60 min, meeting the requirements of the mineral-phase ratio of high-belite Portland cement clinker. Microscopic image analysis (Figure 18) further identified numerous voids and the incomplete development of small crystal particles at a sintering time of 30 min. This observation highlights that 60 min is the optimal sintering time, as it provides the most balanced mineral-phase composition while minimizing issues related to incomplete crystal formation.

**Figure 19:**
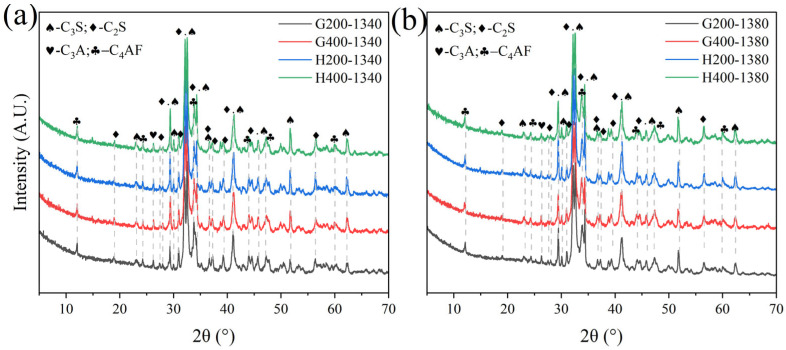
XRD of samples G and H calcined with limestone feedstocks of varying fineness.

**Figure 20:**
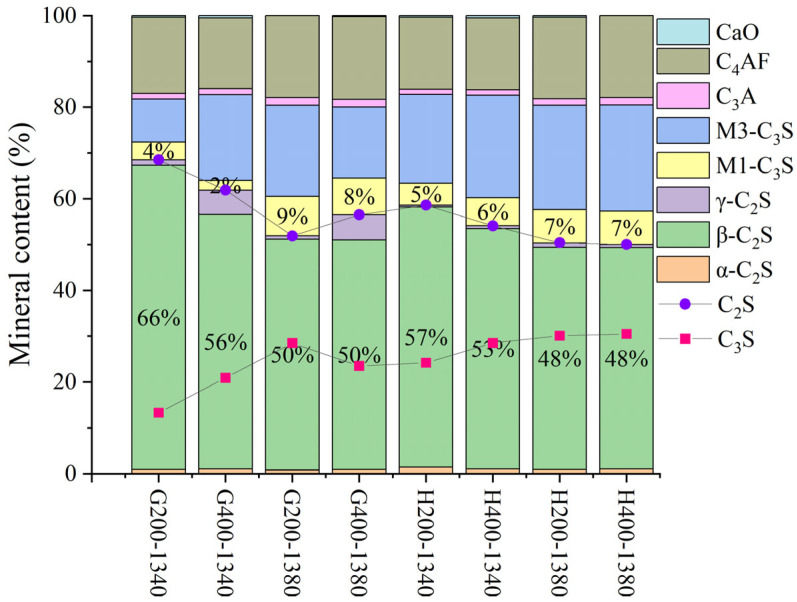
The mineralogical composition of samples H and G following calcination with limestone feedstocks of varying fineness.

Due to the significant SiO_2_ content in LGL, its crystallinity can adversely affect clinker properties. To address this issue, the limestone was classified and ground into various particle sizes, followed by sieving through 200-mesh and 400-mesh square-hole sieves to ensure uniform fineness before batching for clinker preparation under identical calcination conditions. XRD analysis indicated that varying limestone grain sizes do not significantly influence the C_4_AF and C_3_A mineral-phase contents in Figure 19 and Figure 20. For sample H, the mineral composition remains consistent when using 400-mesh sieved limestone, whereas for sample G, the effect of limestone fineness is more pronounced due to a higher proportion of limestone in raw meal preparation (11.5% in sample G compared to 10% in sample H). This underscores the importance of controlling the limestone fineness according to specific requirements.

**Figure 21:**
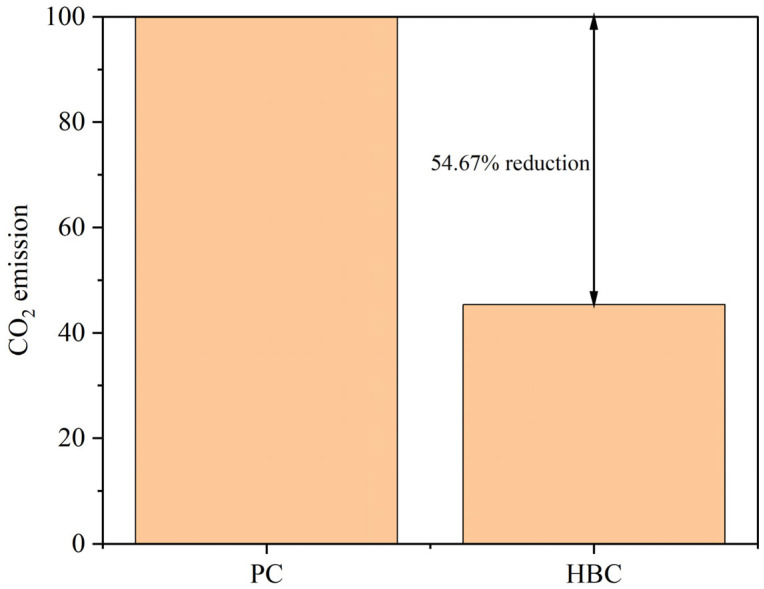
Comparison of CO_2_ emissions between HBC and PC.

In this experiment, utilizing low-grade limestone and calcium carbide slag to replace high-quality limestone for clinker calcination can significantly reduce the CO_2_ emission due to the decomposition of raw materials in the production of cement process. Because the main component of calcium carbide slag is Ca(OH)_2_, CaO and H_2_O are produced during decomposition, and there is no CO_2_ emission; for this reason, according to the TG-DSC curve of sample H, the H_2_O produced during calcination is analyzed to calculate the required CaO, as shown in Equations (4)–(7). And the percentage of CO_2_ reduction is calculated by replacing CaCO_3_, as shown in Figure 21.

(4) $${\mathrm{Ca}(\mathrm{OH})}_{2}\to \mathrm{CaO}+2H_{2}O$$

(5) $${\mathrm{CaCO}}_{3}\to \mathrm{CaO}+{\mathrm{CO}}_{2}$$

(6) $$m_{2CO_{2}}=\frac{11}{9}m_{H_{2}O}$$

(7) $$R_{CO_{2}}=(1-\frac{m_{2CO_{2}}}{m_{1CO_{2}}+m_{2CO_{2}}})\times 100\%$$

Here, $R_{CO_{2}}$ is the proportion of CO_2_ emission reduction, $m_{1CO_{2}}$ is the reduced CO_2_ emissions during calcination, and $m_{2CO_{2}}$ is the CO_2_ emissions from calcination.

## 4. Conclusions

In this study, high-belite Portland cement clinker was prepared using LGL and solid waste materials (calcium carbide slag and steel slag). The effects of varying KH values, calcination temperatures, and sintering times on clinker formation were systematically investigated. Additionally, a preliminary exploration of the influence of fineness of limestone on clinker calcination was conducted. The conclusions drawn from this study are summarized as follows:

High-belite Portland cement was successfully prepared by changing the KH value and calcining system. A too-low or too-high KH will cause the content of C_2_S and C_3_S in the clinker mineral phase to be either too high or too low. High-belite Portland cement clinker with KH = 0.86 was successfully prepared at 1340 °C in 1 h, where the mineral phase of β-C_2_S can reach 56.8%, and the α-C_2_S can be up to 1.4%. The early hydration activity of α-C_2_S is higher than that of β and γ, which can make up for the defect of low early hydration strength of high-belite Portland cement clinker.

In this research, the difference in fineness (6.133 μm and 4.907 μm) of LGL may not affect the mineral composition of the high-belite Portland cement clinker. This may be due to LGL occupying a relatively small proportion of raw material combinations. Further investigations need to be performed.

Overall, the high-belite Portland cement clinker prepared from low-grade limestone and solid wastes can effectively reduce CO_2_ emission. In this study, the CO_2_ emission of the prepared high-belite Portland cement clinker was 54.67% lower than that of ordinary Portland cement clinker.

## Figures and Tables

**Table 1:** Chemical compositions of raw materials (wt.%).

Materials	LOSS	SiO_2_	Al_2_O_3_	Fe_2_O_3_	CaO	MgO	K_2_O	Na_2_O	SO_3_
Low-grade limestone	31.01	19.90	6.49	2.13	36.14	1.88	1.29	0.40	0.17
Calcium carbide slag	25.95	2.90	1.42	0.46	68.50	0.12	0.03	0	0.44
Clay	12.38	63.59	11.45	1.03	6.10	2.24	2.48	0.45	0
Steel slag	−0.70	14.02	4.14	24.35	43.28	5.36	0.02	0.08	0.16

**Table 2:** Composition of clinker ratios.

	Low-Grade Limestone	Calcium Carbide Slag	Clay	Steel Slag	KH	SM	IM
A	16.5	52.5	20	11	0.72	2.4	1.3
B	15	54	20	11	0.74	2.4	1.3
C	13.5	55.5	20	11	0.76	2.4	1.3
D	13	56.75	20	10.25	0.78	2.5	1.3
E	11	58	20	11	0.80	2.4	1.3
F	12.5	58	19	10.5	0.82	2.4	1.3
G	11.5	59	19	10.5	0.84	2.4	1.3
H	10	60	19	11	0.86	2.4	1.2
I	9	61.5	19	10.5	0.88	2.4	1.3
J	7.5	62.5	19	11	0.90	2.4	1.2

## Data Availability

The original contributions presented in this study are included in the article. Further inquiries can be directed to the corresponding authors.
